# Major Shifts in the Spatio-Temporal Distribution of Lung Antioxidant Enzymes during Influenza Pneumonia

**DOI:** 10.1371/journal.pone.0031494

**Published:** 2012-02-15

**Authors:** Yoshiyuki Yamada, Gino V. Limmon, Dahai Zheng, Na Li, Liang Li, Lu Yin, Vincent T. K. Chow, Jianzhu Chen, Bevin P. Engelward

**Affiliations:** 1 Interdisciplinary Research Group in Infectious Diseases, Singapore-Massachusetts Institute of Technology Alliance in Research and Technology, Singapore; 2 Department of Microbiology, National University of Singapore, Singapore; 3 Koch Institute for Integrative Cancer Research and Department of Biology, Massachusetts Institute of Technology, Cambridge, Massachusetts, United States of America; 4 Department of Biological Engineering, Massachusetts Institute of Technology, Cambridge, Massachusetts, United States of America; Commissariat a l'Energie Atomique(cea), France

## Abstract

With the incessant challenge of exposure to the air we breathe, lung tissue suffers the highest levels of oxygen tension and thus requires robust antioxidant defenses. Furthermore, following injury or infection, lung tissue faces the additional challenge of inflammation-induced reactive oxygen and nitrogen species (ROS/RNS). Little is known about the identity or distribution of lung antioxidant enzymes under normal conditions or during infection-induced inflammation. Using a mouse model of influenza (H1N1 influenza virus A/PR/8/34 [PR8]) in combination with bioinformatics, we identified seven lung-abundant antioxidant enzymes: Glutathione peroxidase 3 (Gpx3), Superoxide dismutase 3 (Sod3), Transferrin (Tf), peroxyredoxin6 (Prdx6), glutathione S-transferase kappa 1 (Gstk1), Catalase (Cat), and Glutathione peroxidase 8 (Gpx8). Interestingly, despite the demand for antioxidants during inflammation, influenza caused depletion in two key antioxidants: Cat and Prdx6. As Cat is highly expressed in Clara cells, virus-induced Clara cell loss contributes to the depletion in Cat. Prdx6 is also reduced due to Clara cell loss, however there is a coincident increase in Prdx6 levels in the alveoli, resulting in only a subtle reduction of Prdx6 overall. Analogously, Gpx3 shifts from the basement membranes underlying the bronchioles and blood vessels to the alveoli, thus maintaining balanced expression. Taken together, these studies identify key lung antioxidants and reveal their distribution among specific cell types. Furthermore, results show that influenza depletes key antioxidants, and that in some cases there is coincident increased expression, consistent with compensatory expression. Given that oxidative stress is known to be a key risk factor during influenza infection, knowledge about the antioxidant repertoire of lungs, and the spatio-temporal distribution of antioxidants, contributes to our understanding of the underlying mechanisms of influenza-induced morbidity and mortality.

## Introduction

With its approximately 70 m^2^ of surface area in direct contact with the air we breathe, lung tissue needs to have a particularly robust antioxidant system. Furthermore, during inflammation reactive oxygen and nitrogen species (ROS/RNS), such as superoxide, hydrogen peroxide, nitric oxide (NO) and peroxynitrite, can cause additional stress by directly or indirectly breaking covalent bonds in DNA, proteins and lipids [1]. To keep oxidative and inflammation-driven stress in check, cells exploit both by non-enzymatic antioxidants, including glutathione and ascorbic acid, and enzymatic antioxidants, such as superoxide dismutase (Sod), catalase (Cat), glutathione peroxidase (Gpx) and peroxiredoxin (Prdx) [2]. While normally these defenses suffice to protect the lungs against oxidative stress, during infections such as influenza, there can be a loss of balance between pro-oxidants and antioxidants, causing potentially lethal conditions [3], [4]. Despite the importance of oxidative stress in disease progression during influenza, relatively little is known about the identities of the key antioxidant enzymes in the lung, the cell types in which they are expressed, or their dynamics following infection.

Superoxide is one of the most abundant ROS, being formed at high levels by immune cells in response to infection. Superoxide and hydrogen peroxide (a byproduct of superoxide), are effective toxicants against invading microbes, but their levels must be kept in check in order to suppress collateral damage to normal tissues. To defend normal tissues against superoxide and hydrogen peroxide, several antioxidant enzymes work in concert. Sod serves to catalyze a rapid conversion of superoxide into hydrogen peroxide, which is then detoxified by downstream antioxidant enzymes. There are two types of intracellular Sod, Cu,Zn-SOD (Sod1) in the cytoplasm and Mn-SOD (Sod2) in the mitochondrion. In addition, high levels of EC-SOD (Sod3) are located on the external surfaces [5]. Hydrogen peroxide is then decomposed into H_2_O by downstream antioxidant enzymes, including Cat, Gpx, Prdx and many other peroxidases [2]. It is therefore the coordinated action of these enzymatic antioxidant enzymes that is necessary in order to assure maintenance of a balance between pro-oxidants and antioxidants, thus preventing oxidative stress.

During influenza-induced inflammation, immune cells produce superoxide and NO by activation of xanthine oxidase and inducible nitric oxide synthase (iNOS) as antimicrobial responses. The resulting high levels of reactive oxygen and nitrogen species lead to a reduction in the levels of ascorbic acid and reduced glutathione [3], [6]. In response, enzymatic defenses including heme oxygenase 1 (Hmox1), Gpx1, and thioredoxin reductase 1 can be induced during influenza infection [7], [8]. Nevertheless, pro-oxidants as well as antioxidants are needed, since elevated levels of NO-producing macrophages and dendritic cells ultimately help to control morbidity and mortality associated with highly pathogenic influenza A viruses [4].

Influenza-induced oxidative stress can lead to catastrophic loss of cell and tissue function. As little is known about that spatio-temporal distribution of antioxidants, here, we identified and traced the levels of the major lung antioxidants through the course of disease. Interestingly, we observe that antioxidant levels not only rise for some enzymes and fall for others, but furthermore there are significant shifts in the regions and cell types in which they are expressed. Indeed, for two antioxidants, viral-induced depletion in one cell type is associated with increased expression in another, suggesting compensatory expression. Given that appropriate antioxidant responses govern cell survival, knowledge of the coordinated action of antioxidant enzymes contributes to our understanding of disease susceptibility and progression.

## Results

### Bioinformatic analysis reveals the spectrum of antioxidants expressed at high levels in lung cells, immune cells, and stem cells

Our first objective was to identify lung-abundant, and possibly lung-specific, antioxidant enzymes. We therefore carried out a bioinformatics analysis of published mouse gene expression data (GNF Mouse GeneAtlas V3, GSE10246, [9]) using Gene Spring GX software (Agilent Technologies). Gene expression of 34 antioxidant enzymes (Table 1) that are present in the lung were compared to non-lung samples, which includes 90 different organs, tissues and cells. Antioxidants with more than a 5-fold increase in the lung relative to non-lung samples were classified to be lung-abundant. Although there was no antioxidant proteins exclusively expressed in the lung, we nevertheless identified seven key lung-abundant antioxidant enzymes: Gpx3 (35.97-fold), Sod3 (32.04), transferrin (Tf, 17.58), Prdx6 (11.39), glutathione S-transferase kappa 1 (Gstk1, 6.55), Cat (6.06) and Gpx8 (5.88) (Table 1). Identification of lung-abundant antioxidants enables more detailed studies of their spatio-temporal distribution following infection.

**Table 1:** Summary of lung-, immune cell- and stem cell-abundant antioxidants elucidated from published gene expression data GSE10246.

Abundant in	Gene*		Probe ID	Fold increase**		Subcellular localization***
	Symbol	Entrez ID				
**Lung**	Gpx3	14778	1449106_at	35.97		EC, PL
	Sod3	20657	1417633_at	32.04		EC, PL
	Tf	22041	1425546_a_at	17.58		EC, PL
	Prdx6	11758	1423223_a_at	11.39		Cyt, Lys, Cv
	Gstk1	76263	1452823_at	6.55		Mit
	Cat	12359	1416430_at	6.06		Per
	Gpx8	69590	1424099_at	5.88		Mem
**Immune cells**						
LPS-activated macrophage	Hmox1	15368	1448239_at	114.74	(24hrs post activation)	ER, Mic
(Bone marrow)	Ptgs2	19225	1417262_at	154.01	(6 hrs post activation)	Mem
	Sod2	20656	1417193_at	11.91	(6 hrs post activation)	Mit
Mast cells	Ptgs1	19224	1436448_a_at	75.01	(+IgE)	Mem
**Stem cells & Progenitors**						
Granurocytes	Mpo	17523	1415960_at	363.11	(Progenitors)	Lys
Dendritic cells	Ptgs2	19225	1417262_at	18.83	(CD8-)	Mem
Bone marrow	Mpo	17523	1415960_at	299.11		Lys
	Epx	13861	1449136_at	67.44		Cyt
Common myeloid progenitors	Mpo	17523	1415960_at	203.73		Lys
Stem cells HSC	Mpo	17523	1415960_at	75.96		Lys
Emryonic stem cell line V26	Ptgs2	19225	1417262_at	11.81		Mem

*Antioxidant genes that are not abundant in the above tissue and cells: Duox1, Gpx1, Gpx2, Gpx4, Gpx5, Gpx6, Gpx7, Gsr, Lpo, Prdx1, Prdx2, Prdx3, Prdx4, Prdx5, Prdx6-rs1,Sod1, Srxn1, Tpo, Txnrd1, Txnrd2, Txnrd3.

**Fold increase against the base line of all other tissues and cells in GSE10246.

***Abbreviations: Cyt, cytoplasm; Cv, cytoplasmic vesicle; EC, extracellular; ER, endoplasmic reticulum; Lys, lysosome; Mit, mitochondrion matrix; Mem, membrane; Per, peroxisome; PL, plasma.

A complicating factor when studying the prevalence of different antioxidant enzymes is the potential contribution of antioxidants present within infiltrated immune cells and progenitors during inflammation and tissue repair, respectively. We therefore assessed expression of antioxidant genes in key immune cells, including macrophages, granulocytes, dendritic cells, NK cells, B cells, T cells, thymocytes and mast cells, as well as progenitors and stem cells. We found that immune cells, progenitors, and stem cells express lower levels of the lung-abundant antioxidant transcripts than lung tissue. For example, the range of expression was: Gpx3 (−10.42 to −2.02 fold, except for 3.33 in bone marrow), Sod3 (−3.52 to −1.68) and Tf (−14.35 to 3.79, except for 4.55 in bone marrow). These findings suggest that analysis of the seven lung-abundant antioxidant enzymes during influenza infection will reveal predominantly expression in somatic lung tissue.

Although analysis of normal immune cells did not reveal high levels of expression of lung-abundant antioxidants, it was also possible that cytokines and chemokines present during inflammation might cause macrophages, and other immune cells, to induce antioxidants. Indeed, further analysis of previously published microarray data revealed that some antioxidants, especially prostaglandin-endoperoxide synthase 2 (Ptgs2) and Hmox1, are transiently-expressed at very high levels in LPS-activated bone marrow macrophages with the peaks at 6 and 24 h post-activation, respectively (Table 1). Additionally, we found that myeloperoxidase (Mpo) is expressed at high levels in neutrophil progenitors. In contrast, all other differentiated immune cells, progenitors and stem cells express antioxidants at baseline or lower levels compared to lung tissue. Therefore, while there are several noteworthy examples of antioxidants induced in immune cells, the seven lung-abundant antioxidant enzymes are clearly enriched in normal lung tissue.

### Animal model for H1N1 infection

**Figure 1:**
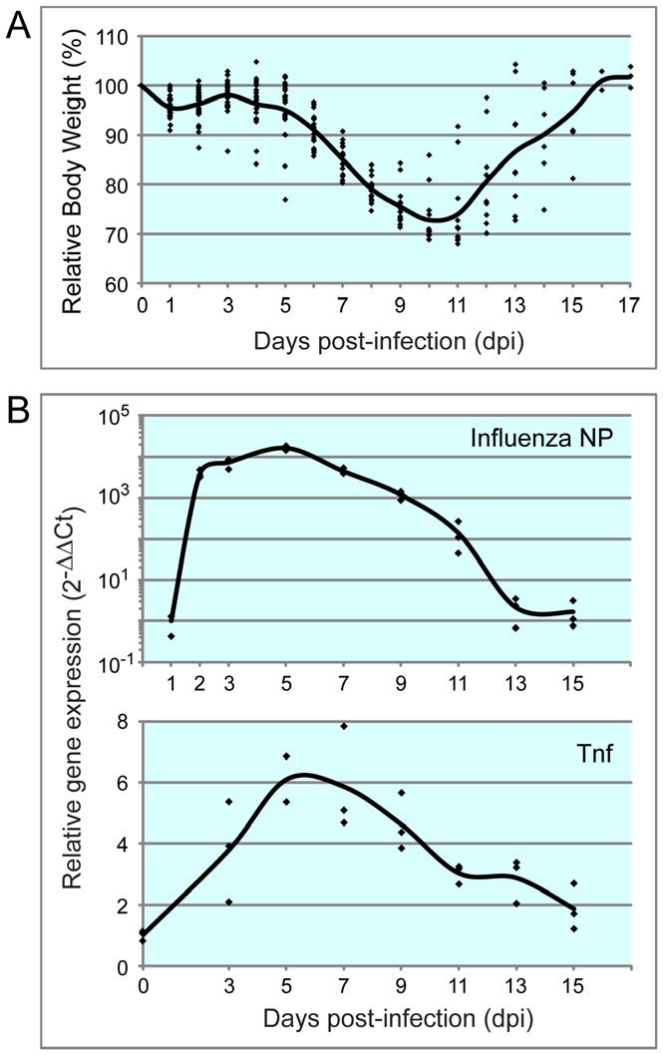
Animal model for H1N1 infection. Mice were infected with sub-lethal dose of PR8 by intra-tracheal inhalation. **A.** Influenza-induced weight loss of the infected mice started from 4 dpi, reached to the peak at 10 and 11 dpi, after which, body weight recovered gradually. Body weights of all infected mice were measured daily. Data of individual mouse and an average of each time point were plotted with rhombus and line, respectively. **B**. mRNA of influenza nucleoprotein (NP) in the lung tissue reached to a maximum at 5 dpi, and decreased by 13 dpi. In contrast, tumor necrosis factor (TNF) expression was the highest around 5–7 dpi. mRNA expression in the lung tissue was investigated by qRT-PCR. Beta-actin was used for normalization.

To study influenza in an animal infection model, mice were infected with a sublethal dose of H1N1 influenza virus A/PR/8/34 (PR8) and monitored over the course of several weeks. Key markers of infection included weight loss, viral load and expression of tumor necrosis factor (Tnf). Influenza-infected animals experienced significant weight loss starting about 5 days after infection, and animals reached their lowest weights at about 10 to 11 dpi, after which, body weights recovered gradually (Fig. 1A). To monitor viral proliferation, expression of influenza nucleoprotein (NP) mRNA was investigated. Viral abundance rose quickly between 1 and 5 dpi and then slowly declines, virtually disappearing by day 13 (Fig. 1B). We also observed an increase in the levels of Tnf, a pro-inflammatory cytokine produced following virus infection with a peak between 5–7 dpi (which is consistent with previous studies [10], Fig. 1B).

### Kinetics of lung-abundant antioxidants following PR8 infection

**Figure 2:**
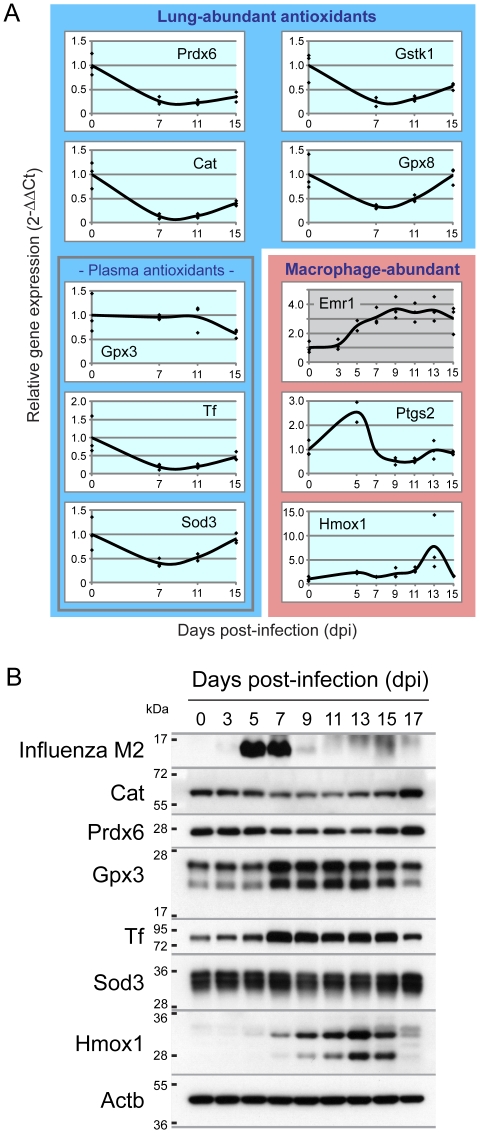
Kinetics of lung-abundant antioxidants following PR8 infection. Mice were infected with sub-lethal dose of PR8 by intra-tracheal inhalation. **A.** Influenza-induced change of transcript levels of the seven lung-abundant antioxidants (in blue box), macrophage cell marker (Emr1 in red box) and activated macrophage-abundant antioxidants (in red box) in the lung tissue was investigated by qRT-PCR. Beta-actin was used for normalization. **B.** PR8 infection reduced lung-abundant Cat and Prdx6, while lung-abundant plasma antioxidants were increased (Gpx3 and Tf) or consistent (Sod3) over the course of infection. Activated macrophage-abundant Hmox1 increased following the clearance of viruses. Lung protein extracts from three mice were pooled, separated by SDS-PAGE and labeled with the indicated antibodies.

To learn about the spatio-temporal dynamics of the seven lung-abundant antioxidants during the course of influenza infection, we first quantified transcript levels via real time RT-PCR (qRT-PCR). Despite the increased demand for antioxidant activity during inflammation, we observed a marked decrease in transcript levels at 7 dpi (immediately after the peak of viral load) for all lung-abundant antioxidants, except for Gpx3 (Fig. 2A, blue box). In contrast, for antioxidants induced in immune cells (red box, Fig. 2A), we observed increased levels of transcripts. Specifically, at 5 dpi (Ptgs2) and 13 dpi (Hmox1) increase, which is consistent with an increase in the macrophage marker, EGF-like module containing, mucin-like, hormone receptor-like 1 (Emr1), (Fig. 2A in red box).

Of particular interest is the observation that antioxidant levels are apparently reduced during infection. To learn if there is also suppression of lung-abundant antioxidants at the protein level, we performed immunoblotting. Initial analysis of viral burden shows that the influenza integral membrane protein M2 protein was detectable 3–9 dpi, peaking on 5 dpi (Fig. 2B and supporting figure S1). Analysis of two of the lung-abundant antioxidants, Cat and Prdx6, revealed a decrease in protein levels (between 7 and 15 dpi), which is consistent with the results for the transcript levels. However, a more subtle reduction was observed for Prdx6 (Fig. 2B and S1). In contrast, extracellular lung-abundant Gpx3 and Tf (but not Sod3), were significantly increased, although transcript levels were not (Fig. 2A). Finally, consistent with the presence of influenza-induced inflammation, the levels of Hmox1 (abundant in LPS-activated macrophage) also increased with a peak at 13 dpi, that is consistent with the observed increase in transcript levels (Fig. 2B and S1).

### Clara and AT2 cells are the major PR8-permissive cell types in mouse lung

**Figure 3:**
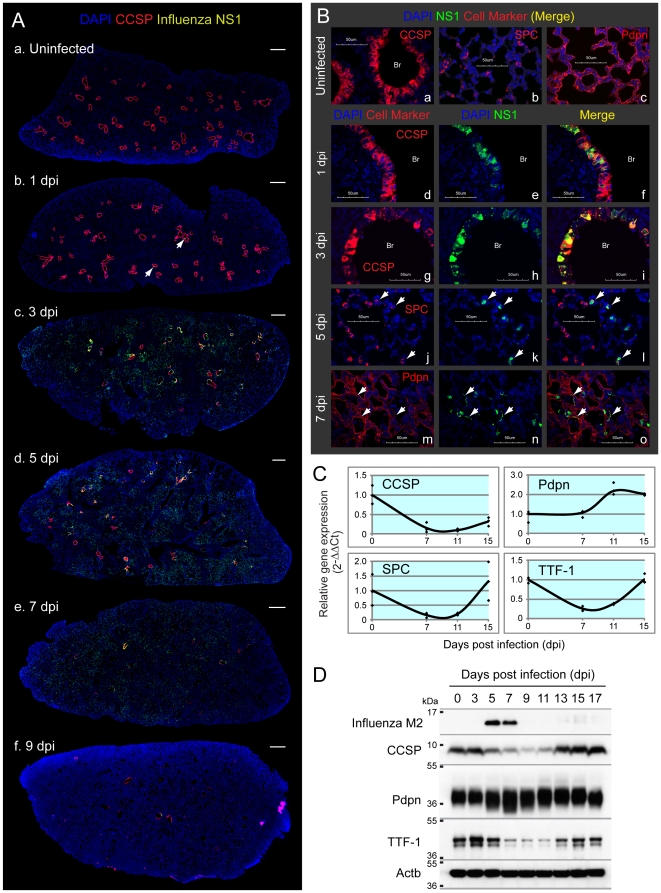
Clara and AT2, but not AT1 cells, are the major PR8-permissive cell types in mouse lung. Mice were infected with sub-lethal dose of PR8 by intra-tracheal inhalation. **A.** PR8 replication sites (viral nonstructural protein 1 [NS1]-positive cells) spread from bronchial cells to alveoli with the peak at 5 dpi and almost completely disappeared by 9 dpi. Lung sections were immunostained with CCSP, NS1 and Prdx6. Slides were scanned by MIRAX MIDI system with the exact same exposure times for DAPI, CCSP, Prdx6 and NS1, respectively, and images were shown with the same adjustment of brightness and contrast. Scanned image for Prdx6 was shown in Fig. 5A. The scale bars represent 500 µm. Infected cells at 1 dpi were pointed with arrows. **B.** NS1 was mainly detected in bronchial (Br) Clara cells at 1 dpi (d, e, f), and then a layer of infected Clara cells lysed at 3 dpi (g, h, i). Alveolar infected cells at 5 dpi were co-stained with SPC (white arrows in j, k, l). NS1-positive AT1 cells (white arrows in m, n, o) were sometimes observed at mildly inflamed area at 7 dpi. Lung sections were immunostained with the indicated antibodies and observed at ×60 magnification with confocal microscope. The scale bars represent 50 µm. **C.** Gene expression of CCSP, SPC and TTF-1, but not Pdpn, was reduced following infection. mRNA expression in the lung tissue was investigated by qRT-PCR. Beta-actin was used for normalization. **D.** PR8 infection reduced protein levels of CCSP and TTF-1, but not Pdpn. Lung protein extracts from three mice were pooled, and were subjected to immunoblotting analysis with the indicated antibodies.

One possible explanation for the reduction in Cat and Prdx6 is that cells that normally express these antioxidants are cleared due to viral infection. To visualize how viral infection spread within the mouse lungs, we first immunostained lung sections with antibodies against viral-nonstructural protein 1 (NS1). Antibody against Clara cell secretory protein (CCSP) was used to distinguish bronchioles from alveoli. We observed that some bronchial cells are NS1-positive at 1 dpi (arrows in Fig. 3A b) and that the majority of lungs are normal. At 3 dpi, viral replication sites spread to most of the bronchial cells and also to several alveolar regions (c). Subsequently, NS1-positive cells were mainly observed in the alveoli (d, e) with a peak at 5 dpi and drastically reduced by 9 dpi (f). These data are in agreement with expression kinetics of NP mRNA (Fig. 1B) and M2 protein (Fig. 2B and S1).

To reveal which cell types are most susceptible to the PR8 infection, we further immunostained tissue sections with antibodies that are specific for the two pneumocytes: alveolar epithelial type 1 (AT1) cells (podoplanin; Pdpn, Fig. 3B c) and alveolar epithelial type 2 (AT2) cells (surfactant protein C; SPC, Fig. 3B b). Analysis of lung tissue on 1 dpi revealed the presence of NS1 protein in bronchial Clara cells (Fig. 3B d, e, f). Virus-infected Clara cells did not change CCSP expression at this timing. The infected Clara cell layer was subsequently destroyed by the virus (Fig. 3B g, h, i) and the virus appears to then spread to cells to the alveoli (Fig. 3A c). At 5 dpi, infected cells co-stained with NS1 and a marker for AT2 cells (SPC; white arrows in j, k, l), indicating that AT2 cells are infected after Clara cells. The widespread disappearance of the AT2 marker indicates that Clara cells are cleared by the virus (Fig. 3B j). In contrast to Clara and AT2 cells, we observed relatively few virus-infected AT1 cells (white arrows in m, n, o) at 7 dpi. Taken together, viral infection primarily leads to rapid clearance of Clara cells, followed by infection and destruction of AT2 cells, which is consistent with previous studies [11].

To learn more about cell-level dynamics, we assessed the relative abundance of key lung cell types using qRT-PCR. In addition to quantifying transcripts specific for Clara, AT2 and AT1 (e.g., CCSP, SPC and Pdpn), thyroid transcription factor-1 (TTF-1) was analyzed, as it is specifically highly expressed in both Clara and AT2 cells in the mouse lung [12], and it regulates CCSP and SPC expression [13], [14]. As shown in Fig. 3C, mRNA of CCSP, SPC and TTF-1 (Clara and AT2 cells) were all significantly reduced during the course of infection, which is consistent with the data showing viral clearance of Clara and AT2 cells. In contrast, there was no significant change in Pdpn (AT1 cells), as expected given the low level of infection of AT1 cells. Consistent with the transcript levels, immunoblotting shows a dramatic decline in the protein levels of key Clara and AT2 cell markers (CCSP and TTF-1 [SPC was not included because they detected as multiple cleaved bands]), but not for AT1 cells (Pdpn), (Fig. 3D and S1). Taken together, Clara and AT2 cells, which harbor the greatest viral load, are also the most depleted from the infected lungs.

### Lung-abundant Cat and Prdx6 are strongly expressed by Clara and AT2 cells in the mouse lung

**Figure 4:**
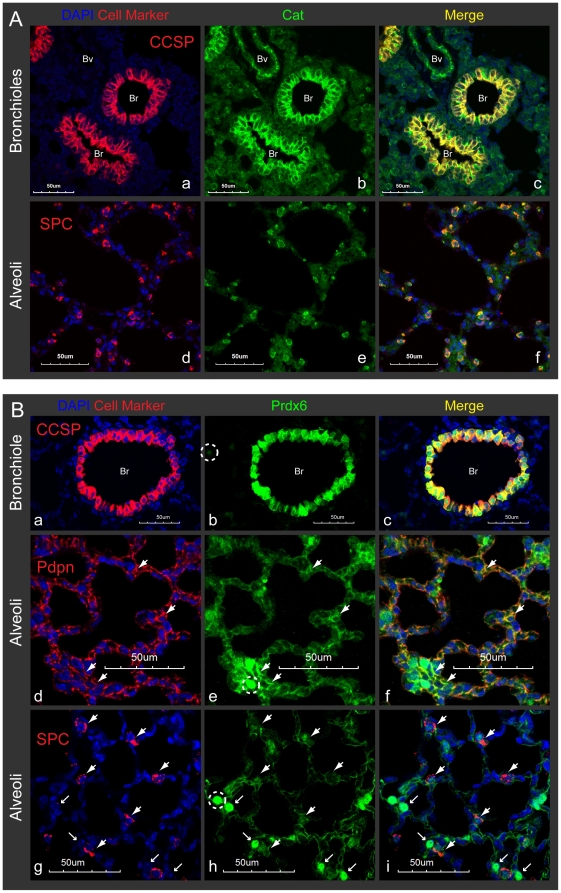
Lung-abundant Cat and Prdx6 are strongly expressed by Clara and AT2 cells in the mouse lung. Lung sections from uninfected mice were immunostained with the indicated antibodies and observed at ×60 magnification with confocal microscope. The scale bars represent 50 µm. Abbreviation: Br; bronchioles, Bv; blood vessels. **A.** Cat was strongly expressed in Clara cells, arterial blood vessels and cells scattered in alveoli (a, b, c). Cells with higher Cat expression at alveoli were co-stained with SPC (d, e, f). **B.** Prdx6 expression was extremely high in bronchial Clara cells (a, b, c). Alveolar Prdx6 was detected in AT1 cells (thick arrows in d, e, f), unknown cell types with Prdx6-positive nuclei (thin arrows in g, h, i) or weakly in AT2 cells (thick arrows in g, h, i). Different exposure settings for image capturing were used for Prdx6 here (Corresponding Prdx6-positive nuclei were circled with dashed line in b, e, h).

To test the possibility that depletion of PR8-permissive cells leads to a change in antioxidant levels, we set out to identify antioxidants that are differentially expressed in Clara and AT2 cells by immunohistochemistry. Cat was strongly detected in Clara cells, with some expression in arterial blood vessels and cells scattered around the alveoli (Fig. 4A a, b, c). Cat was also highly expressed in AT2 cells in alveoli (positive for SPC; Fig. 4A d, e, f). Thus, Cat expression is highest in the two cell types that are most susceptible to viral infection. On the other hand, Prdx6 levels were extremely high in Clara cells and detected in both nucleus and cytoplasm (Fig. 4B a, b, c), whereas expression in AT1 (Pdpn positive) alveolar cells was largely diffuse (thick arrows in d, e, f). We also observed Prdx6-positive nuclei in the alveoli that could potentially be nuclei of AT1 cells (thin arrows in g, h, i and S2A). In rat, lung Prdx6 are strongly expressed in Clara (except for major bronchi) and AT2 cells in the tissue sections and in the cytoplasm of freshly isolated AT2 and alveolar macrophages [15]. However, cells with Prdx6-positive nucleus were not co-stained with SPC in mice (AT2 cells, g, h, i). We also observed that CD68-positive macrophages are scattering in the normal alveoli and sometime at bronchiole (frozen sections were stained with CD68 antibody [Abcam ab53444], Data not shown), however, we unable to detect macrophage like cells which are Prdx6-positive in both nucleus and cytoplasm. Overall, these data show that both Clara and AT2 cells express high levels of Cat, and Clara cells express high levels of Prdx6, such that viral destruction of Clara and AT2 cells is expected to reduce the reserves of these two important antioxidants, which was indeed observed (Fig. 2B).

### Loss of Prdx6-expressing Clara cells coincides with induction of Prdx6 in AT1 cells

**Figure 5:**
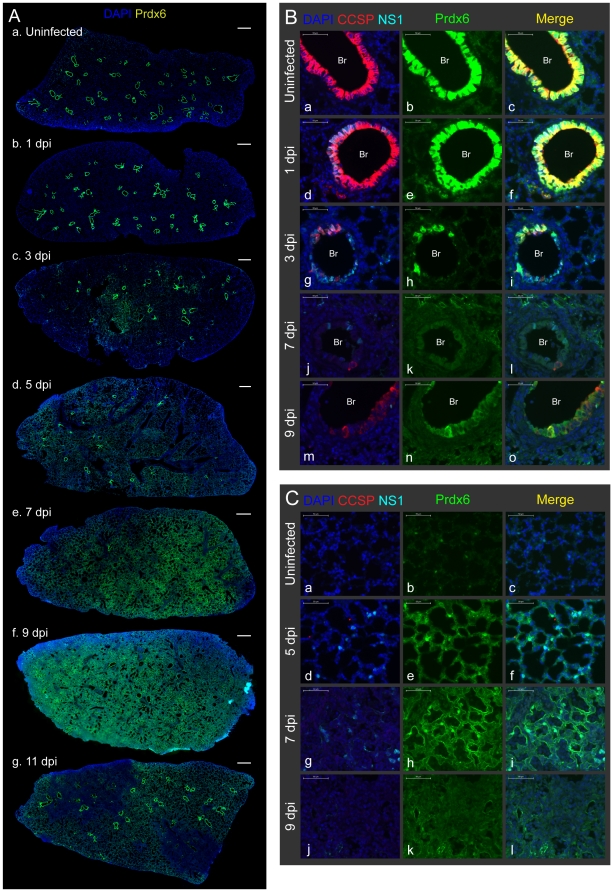
Loss of Prdx6-expressing Clara cells coincides with induction of alveolar Prdx6. Lung sections were immunostained with CCSP, NS1 and Prdx6 antibodies. Slides were scanned by MIRAX MIDI system with the exact same exposure times for DAPI, CCSP, Prdx6 and NS1, respectively, and images were shown either with the same adjustment of brightness and contrast (**A**) or without any adjustments (**B, C**). The scale bars represent 500 µm (**A**) or 50 µm (**B, C**), respectively. **A.** Loss of bronchial Prdx6 coincides with induction of alveolar Prdx6 in the mouse lungs following influenza infection. Scanned image for CCSP and NS1 were shown in Fig. 3A. **B.** Prdx6 in CCSP-positive bronchial (Br) Clara cells drastically decreased following infection (a to l), but started to regenerate at 9 dpi (m, n, o). **C.** Alveolar Prdx6 was increased when many Clara cells were depleted from the lung at 5 dpi (d, e, f), and was strongly expressed at 7 dpi (g, h, i). Prdx6 was then diffused at heavily inflamed area of the alveoli (j, k, l).

Despite the high levels of Prdx6 expression in Clara cells and their subsequent virus-induced clearance, we observed only a slight reduction in the overall levels of Prdx6 (Fig. 2B). To address the question as to why a large change in Clara cell abundance did not result in a coincident large change in the overall levels of Prdx6, we monitored the levels of Prdx6 by immunostaining over the course of the infection. Surprisingly, our staining demonstrated that Prdx6 expression in the lungs is indeed drastically changed following the influenza infection (Fig. 5A). At 1 dpi, there was no change in Prdx6 and CCSP expression in NS1-positive Clara cells (Fig. 5B d, e, f), which suggests that influenza replication does not directly impact the expression of these proteins. Subsequently, however, there was a significant loss of Clara cells (see FIg. 3A), and with a concomitant reduction in staining for bronchial Prdx6 (Fig. 5A c, d and 5B g, h, i, j, k, l). Regeneration of a layer of Clara cells was relatively fast, and the expression level of bronchial CCSP and Prdx6 started to recover after 9 dpi (Fig. 5A f, g, 5B m, n, o and S2B).

**Figure 6:**
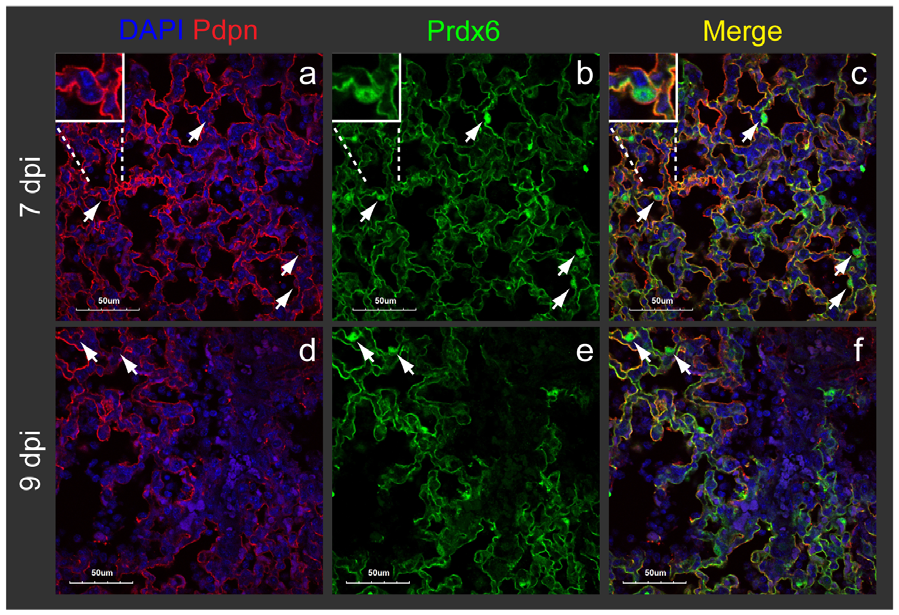
AT1 cells account for a possible compensatory induction of Prdx6. Pdpn and alveolar Prdx6 were exactly co-localized at 7 dpi (a, b, c). Pdpn and Prdx6 were both depleted at heavily inflamed alveoli at 9 dpi (d, e, f). White arrows indicate cells with Prdx6-positive nucleus. Immunostained slides were observed at ×60 magnification in the confocal microscope. The scale bars represent 50 µm.

**Figure 7:**
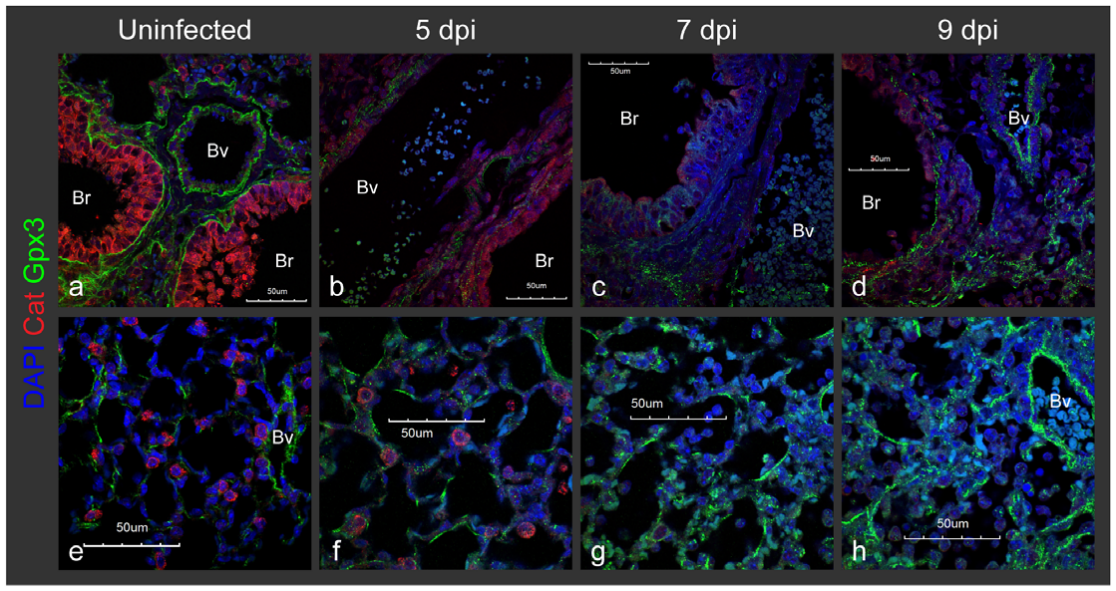
Lung Gpx3 shift from the basement membrane to the alveolar space following infection. Gpx3 at basement membrane of blood vessels (Bv) and bronchioles (Br) was totally diffused at 5 and 7 dpi (a, b, c), but started to accumulate again at 9 dpi (d). In contrast, Gpx3 gradually increased and accumulated in the alveolar spaces (e, f, g, h). Mice were infected with sub-lethal dose of PR8 by intra-tracheal inhalation. Lung sections from uninfected mice were immunostained with the indicated antibodies and observed at ×60 magnification with confocal microscope. The scale bars represent 50 µm.

In contrast to Clara cells, a very different picture emerges for the alveolar spaces. As in un-infected mice (5A a and 5C a, b, c), Prdx6 was weakly detected in the alveolar spaces at 1 dpi (5A b). However, by 5 dpi, there is a significant increase in the overall levels of Prdx6 expression (5A d and 5C d, e, f). Expression of alveolar Prdx6 reached to the peak at 7 dpi (5A e and 5C g, h, i) and diffused at 9 dpi (5A f and 5C j, k, l). Given that Prdx6 exactly co-localized with Pdpn (Fig. 6 a, b, c), it appears that AT1 cells account for a possible compensatory induction of Prdx6. Interestingly, strong induction of Prdx6 in AT1 cells is a transient event and AT1 cells in the inflamed alveoli were depleted after 9 dpi (Fig. 6 d, e, f, and S2B). At 11 dpi, Prdx6 in heavily inflamed alveoli totally disappeared, but the marked regeneration of Prdx6 was observed in the bronchioles (Fig. 5A g). As we did not observe Prdx6-positive nuclei in the area where Pdpn (AT1 cells) were depleted (Fig. S2B), Prdx6 is generally expressed by Clara and AT1 cells in the mouse lungs. Unlike Prdx6, Cat was not induced in AT1 cells (see Fig. 7 f, g, h), which is consistent with the observation of an overall decline in Cat. Together, these data reveal a balancing act wherein Prdx6-expressing Clara cell loss is followed by an increase in Prdx6 in alveolar AT1 cells, thus minimizing the overall loss of Prdx6.

### Lung Gpx3 shifts from the basement membrane to alveoli following infection

In healthy mouse lungs, we observed that Gpx3 accumulates at the basement membranes that underlie blood vessels and bronchioles (which is consistent with previous studies [16]; Fig. 7 a and S3A). Additionally, we found a weak association of Gpx3 with other cell types, including AT2 cells, creating a fairly uniform distribution on the surface of alveoli (Fig. 7 e and S3A). These data are consistent with a model wherein Gpx3 is normally secreted from AT2 cells (and other cell types) and targets basement membranes or cell surfaces.

Following influenza infection, Gpx3 became diffuse by 5–7 dpi (Fig. 7 b, c). Ultimately, as tissue recovered from influenza infection, Gpx3 started to accumulate again at the basement membranes 9 dpi (d). In contrast to the observed drop in basement membrane associated Gpx3 during viral replication period (5–7 dpi), the levels of alveolar Gpx3 gradually increased following infection (Fig. 7 e, f, g, h). Alveolar Gpx3 rarely co-localized with Prdx6 in AT1 cells (thick arrows in Fig. S3B a, b, c), showing that Gpx3 is not bound uniformly to the surface of alveolar cells. Note that we also observed the presence of intracellular Gpx3 within infiltrated cells, but the specific cell type is not known (thin arrows in Fig. S3B a, b, c). Thus, there is a significant shift in the localization of Gpx3, from basement membranes to alveolar spaces, which is consistent with compensatory expression and/or movement of plasma. Together with Prdx6, these are two independent examples in which a drop in antioxidants in one cell type (either because of cell loss or because of changes in affinity) is associated with an increase in the same antioxidant in another location.

## Discussion

It is widely recognized that imbalances in the lung antioxidant system can lead to oxidative stress, which in its most severe form, potentiates influenza-induced morbidity and even mortality [3]. Nevertheless, remarkably little is known about the nature of antioxidant enzymes that are most prevalent in the lung, or about how infection impacts the levels of antioxidant enzymes. Based upon published expression profiles, we have identified seven antioxidant proteins that are particularly abundant in the lung and we have traced their spatio-temporal profiles during the course of infection. Interestingly, we observed dramatic shifts in the levels of antioxidants during infection, and we found that some of the most significant changes are due to loss of influenza virus permissive cells, including bronchial Clara cells and alveolar AT2 cells.

To learn about the roles of Clara and AT2 cells in modulating the levels of antioxidant enzymes, we used immunohistochemistry to analyze the antioxidant expression in these cells. Among the 7 key lung-abundant antioxidant enzymes, Clara cells strongly express two intracellular antioxidants, Cat and Prdx6. Clara cells have several functions, one of which is to protect bronchioles by providing a lining of cells and by secreting a variety of proteins, including CCSP and surfactant A (SPA) [17]. In addition, Clara cells also able to break down harmful substances inhaled into the lungs and to detoxify them in smooth endoplasmic reticulum via cytochrome P450 enzymes, which generate ROS [18], [19]. It is thus reasonable to conjecture that high levels of intracellular antioxidant enzymes protect Clara cells against ROS stress that results both from a high oxygen tension and from Clara cell-specific cytochrome P450 enzymes.

Although Prdx6 is robustly expressed in Clara cells and Clara cells become depleted, the overall levels of Prdx6 stay relatively constant. Analysis of the dynamics of Prdx6 during disease progression shows that the levels of Prdx6 rise significantly within the alveolar spaces. These data are consistent with antioxidant rebalancing, or compensatory expression. This response is not driven by viral replication, since Prdx6 induction was observed in both infected and uninfected cells. Importantly, Prdx6 levels rise in AT1 cells, which cover more than 95% of the surface of alveoli and account for gas exchange [20]. Therefore, even a relatively small increase in the levels of Prdx6 when analyzed by immunohistochemistry, can lead to a significant overall increase in the levels of Prdx6 in the lung, thus compensating for the drop in Prdx6 due to the loss of Clara cells. Rat AT1 is known to act not only as a barrier for gas exchange but also as a barrier to oxidative injury via secretion of apolipoprotein E and Tf [21]. Likewise, induction of Prdx6 in mouse AT1 cells may also provide a critical defense against infection-induced oxidative stress.

Prdx6 is a unique bifunctional enzyme with GSH peroxidase activity (which reduces hydrogen peroxide and phospholipid hydroperoxide to oxidized glutathione and water) and phospholipase A2 (PLA_2_) activity, whose products signal a pro-inflammatory response [22], [23]. Its lipid peroxidation-reducing activity enables the resolution of oxidized lipids in the cell membrane, which would otherwise have the capacity to amplify damage through a chain reaction of oxidation and lipid breakdown products. Therefore, Prdx6 has direct protective effect against toxicity and apoptosis [24]. Interestingly, Prdx6 demonstrates lung-specific functions via the interaction with SPA that translocates Prdx6 into lamellar body and extracellular space [25]. In addition, SPA regulates PLA_2_ activity of Prdx6 and alters its ability to degrade the major phospholipid of surfactant [25], [26]. Finally, PLA_2_ activity of Prdx6 also plays a critical role in oxidative stress- and TNF-induced apoptosis [27]. Further studies on the mechanism of Prdx6 induction in AT1 cells and its potential role in suppressing influenza-induced apoptotic cell death are in progress.

After infecting Clara cells, the virus moves deeper into the lung and replicates in AT2 cells, which are found in the alveoli. AT2 cells are known as secretory cells which release the components of surfactant and extracellular matrix [28] and extracelluar antioxidants Gpx3 and Sod3 [16], [29]. These observations are consistent with a role for AT2 cells in providing antioxidant protection to cells coated with AT2-produced surfactant (e.g., AT1 cells). Thus, AT2 cells represent another target cell type that, when virally infected, might lead to loss of extracellular antioxidant enzymes. Unexpectedly, however, we did not observe any effects of AT2 cell depletion on antioxidant protein expression levels in the lungs. In particular, GPX3 was increased at the time of drastic loss of AT2 cells. Although both Sod3 and Gpx3 are secreted by AT2 cells in the lungs, they are also secreted by kidney proximal tubule cells [30], [31]. Immunostained lung sections demonstrated that Gpx3, which is normally at the basement membrane of blood vessels, becomes diffuse and is subsequently increased throughout the alveolar spaces. Therefore, it is possible that plasma Gpx3 gains access to the lungs due to increased permeability during infection. Thus, the reduction in AT2-secreted Gpx3 is likely masked by the influx of antioxidants in the plasma, thus providing alternative mechanism for maintaining the balance of antioxidants during infection.

In summary, here we have identified novel lung-abundant antioxidant enzymes and we have shown that influenza virus infection impacts their spatio-temporal distribution. Both Clara and AT2 cells are the major influenza-permissive cell types in the lungs, and their loss lead to reduction of key lung antioxidants. However, we present two examples wherein loss of expression of antioxidants is balanced by expression by other cell types. Specifically, both the Clara-enriched Prdx6 and the AT2-enriched Gpx3 are maintained, possibly by compensatory expression system during infection. Taken together, this work reveals several novel lung-specific antioxidants and new mechanisms by which the lungs maintain their levels of antioxidants during infection. Influenza toxicity can be caused by oxidative stress, which can be exacerbated by imbalances in the levels of lung-abundant antioxidants. A deeper understanding of the biology and the responses of lung-abundant antioxidants to influenza is therefore fundamental to our understanding of influenza-induced morbidity and mortality.

## Materials and Methods

### Mice and virus

The H1N1 influenza virus A/PR/8/34 strain (PR8) was purchased from American Type Culture Collection (ATCC). PR8 was propagated in embryonated chicken egg at 37°C for 72 h, and the allantoic fluid was harvested as a viral stock. Virus titers were determined by the plaque assay via infection of Madin-Darby Canine Kidney (MDCK) cells. Ten to twelve weeks old female C57/BL6 mice were housed in BSL2 facilities, and infected with sub-lethal dose of PR8 (30 PFU in 75 µl of phosphate buffered saline [pH 7.4] per mouse) by intra-tracheal inhalation after anesthetization. Lungs were harvested from anesthetized mice at indicated time points and stored at −80°C until use. Infected mice did not recover completely at the end of our experiments (17 dpi) as we observed some infiltrated area in the lung sections (Data not shown).

### Ethics Statement

This study was carried out in strict accordance with the with the National Advisory Committee for laboratory Animal Research (NACLAR) Guidelines (Guidelines on the Care and Use of Animals for Scientific Purposes) in facilities licensed by the Agri-Food and Veterinary Authority of Singapore (AVA), the regulatory body of the Singapore Animals and Birds Act. The protocol was approved by the Institutional Animal Care and Use Committee (IACUC), National University of Singapore (Permit Number: IACUC 117/10).

### Antiobodies

Primary antibodies used in this study were shown in supporting table S1. Secondary antibodies were purchased from the following sources: horseradish peroxidase (HRP)-conjugated anti-goat, -mouse and -rabbit secondary antibodies from DAKO; Alexa Fluor 488-, 546 or 647-labeled anti-goat, -mouse, -rabbit and -rat secondary antibodies from Invitrogen.

### Quantitative real-time RT-PCR

Total RNA was extracted by using Qiagen RNeasy mini kit and treated with DNaseI (Qiagen). RNA concentration was measured by the ND-1000 spectrophotometer (NanoDrop Technologies) and 1 µg of RNA were reverse-transcribed by using oligo(dT) and iScript reverse transcriptase (Bio-rad). PCR was performed with the Bio-Rad CFX-96 real-time system using Ssofast Evagreen Supermix according to the manufacturer's instructions (Bio-Rad). Primers used in this study were listed in supporting information (Table S2). Previously reported primer sequences were obtained from PrimerBank (http://pga.mgh.harvard.edu/primerbank/index.html) and a literature [32]. PCR was carried out for 95°C for 30 s, 40 cycles at 95°C for 1 s and 60°C for 10 s. Data were normalized with beta actin. Trends of the changes in gene expression were confirmed by the two independent experiments.

### Immunoblotting

Lungs were lysed with 2× Laemmli sample buffer without bromophenol blue and the protein concentration was determined by the Bio-rad Protein Assay kit. Five µg of protein (10 µg for Hmox1) were separated by SDS-PAGE and transferred to nitrocellulose membranes (Bio-rad). Membranes were incubated with a primary antibody for overnight at 4°C, subsequently with HRP-conjugated secondary antibody for 1 h at room temperature, and detected using the enhanced chemiluminescence (ECL) prime detection reagents (GE healthcare). Trends of the changes in protein expression were confirmed by the two independent experiments.

### Immunofluorescent staining of paraffin sections

Lungs harvested from anesthetized mice were fixed in 10% neutral buffered formalin solution (Sigma Aldrich) overnight, followed by embedding in paraffin with tissue processor (Leica). Five µm sections on poly-L-lysine coated slides (Thermal Scientific) were de-waxed with xylene and rehydrated in water. Antigen retrieval was processed with proteinase K (Sigma Aldrich, 20 mg/ml in 50 mM Tris-HCl, 1 mM EDTA, pH 8.0) at 37°C for 30 min. The sections were incubated with appropriate antibody overnight at 4°C, and stained with secondary antibody for 1 h at room temperature. The slides were mounted with anti-fade reagent with DAPI (Invitrogen) and then scanned by high-resolution MIRAX MIDI system (Carl Zeiss). Images at ×60 magnification were observed under the confocal microscope FluoView FV1000 (Olympus).

## Supporting Information

Figure S1 **Immunoblotting results of the individual mice.** Lung protein extracts from three mice at 0, 7, 9 and 15 dpi were subjected to immunoblotting analysis with the indicated antibodies.
(TIF)

Figure S2 **Prdx6-positive nuclei are not observed in the Pdpn (AT1cells)-negative inflamed area of alveoli.** Immunostained lung sections were scanned by MIRAX MIDI system. **A.** A number of Prdx6-positive nuclei (in green) were scattered in the normal alveoli. **B.** Prdx6-positive nuclei (arrows in b, d, f, h) were not observed in Pdpn-negative inflamed area at 11 dpi (a, c, e). Bronchial Prdx6 started to express strongly at 11 dpi (a, c, e). The section was subsequently stained by H&E staining (g, h). Cluster of five Prdx6-positive nuclei were circled with dashed line. The scale bars represent 100 µm (a, c, e, g) and 50 µm (b, d, f, h), respectively.
(TIF)

Figure S3 **Localization of Gpx3 in both uninfected and infected mice lung.** Mice were infected with sub-lethal dose of PR8 by intra-tracheal inhalation. Immunostained lung sections with the indicated antibodies and observed at ×60 magnification with confocal microscope. The scale bars represent 50 µm. **A.** Gpx3 was abundantly located at the basement membrane of blood vessels (Bv) and bronchioles (Br) in the normal lung. Gpx3 was also weakly detected at alveolus, but did not always appear at the surface of AT2 cells that express higher Cat (white arrows in d, e, f). **B.** Alveolar GPX3 at 7 dpi did not co-localized with AT1 cells that express higher level of Prdx6 (thick arrows in a, b, c). Gpx3 was also strongly detected within some infiltrated cells (thin arrows in a, b, c).
(TIF)

Table S1 **Primary antibodies used in this study.**
(DOC)

Table S2 **Primers used for qRT-PCR in this study.** *Primer sequences were obtained from PrimerBank (http://pga.mgh.harvard.edu/primerbank/index.html).
(DOC)
