# Supplementary figures and images for: Major Shifts in the Spatio-Temporal Distribution of Lung Antioxidant Enzymes during Influenza Pneumonia
Source: PLoS One. 2012 Feb 15;7(2):e31494. doi: 10.1371/journal.pone.0031494 (PMC3280306; doi:10.1371/journal.pone.0031494)

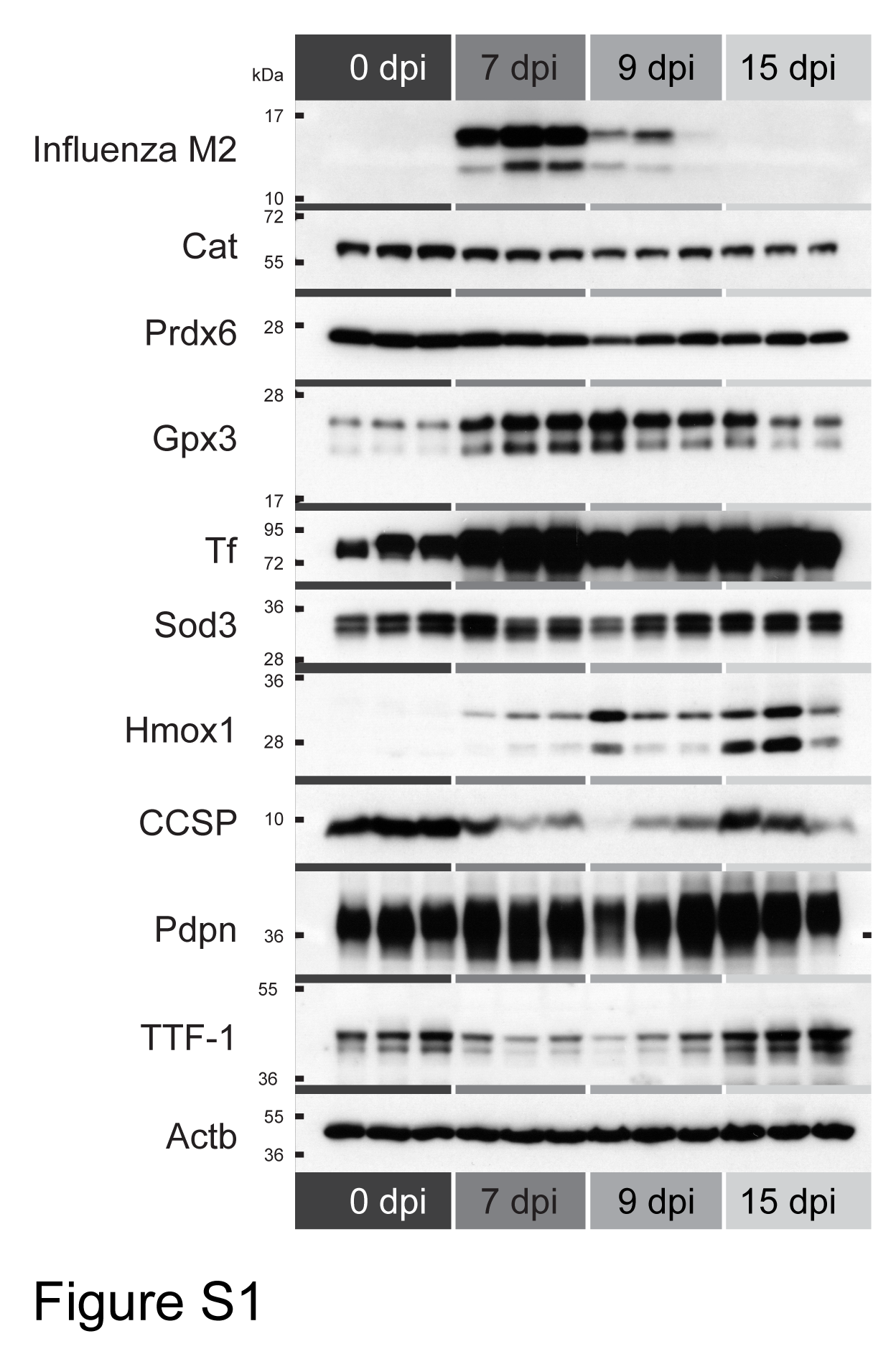

Supplement: Figure S1 — Immunoblotting results of the individual mice. Lung protein extracts from three mice at 0, 7, 9 and 15 dpi were subjected to immunoblotting analysis with the indicated antibodies. (TIF) [file pone.0031494.s001.tif]

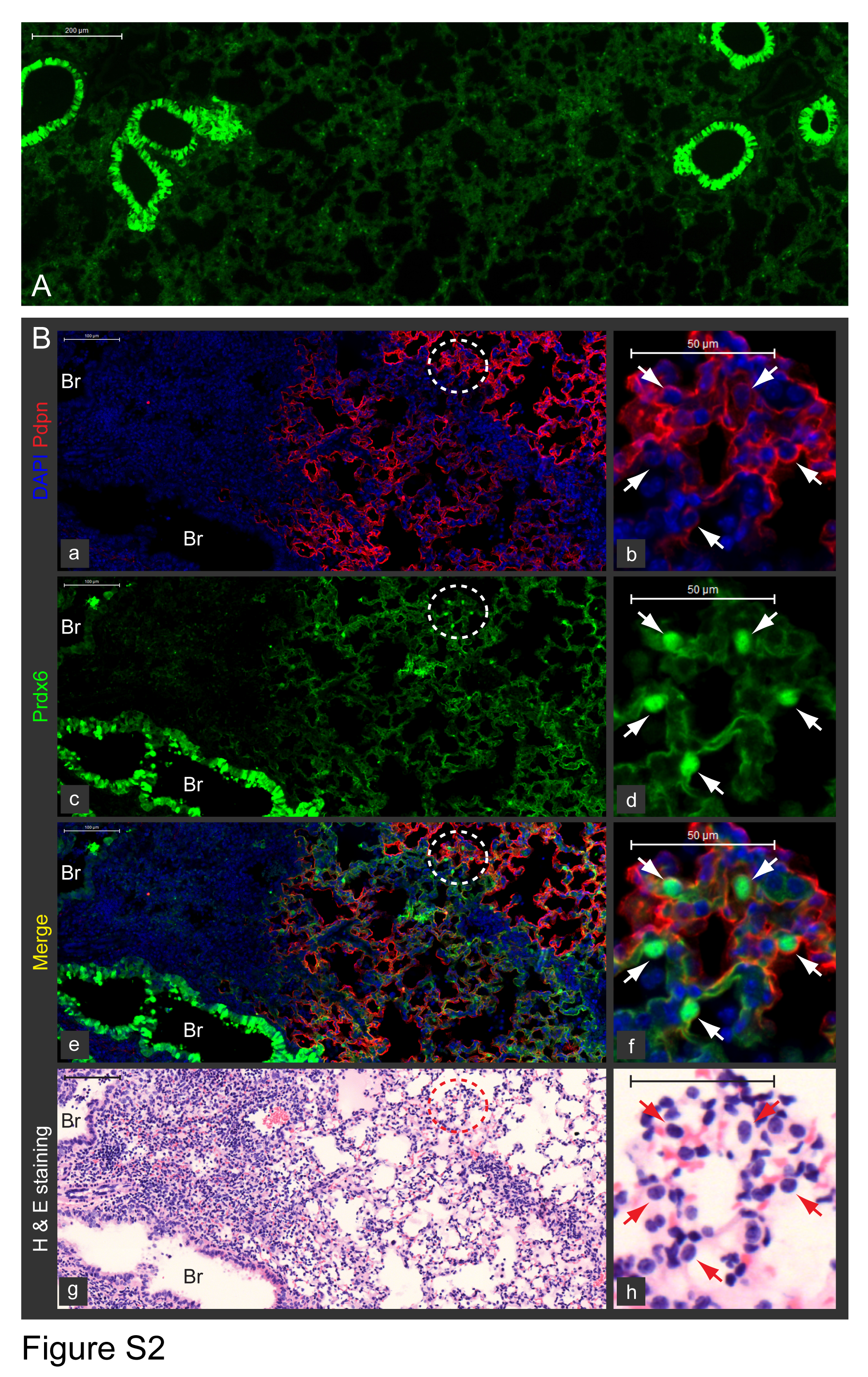

Supplement: Figure S2 — Prdx6-positive nuclei are not observed in the Pdpn (AT1cells)-negative inflamed area of alveoli. Immunostained lung sections were scanned by MIRAX MIDI system. A. A number of Prdx6-positive nuclei (in green) were scattered in the normal alveoli. B. Prdx6-positive nuclei (arrows in b, d, f, h) were not observed in Pdpn-negative inflamed area at 11 dpi (a, c, e). Bronchial Prdx6 started to express strongly at 11 dpi (a, c, e). The section was subsequently stained by H&E staining (g, h). Cluster of five Prdx6-positive nuclei were circled with dashed line. The scale bars represent 100 µm (a, c, e, g) and 50 µm (b, d, f, h), respectively. (TIF) [file pone.0031494.s002.tif]

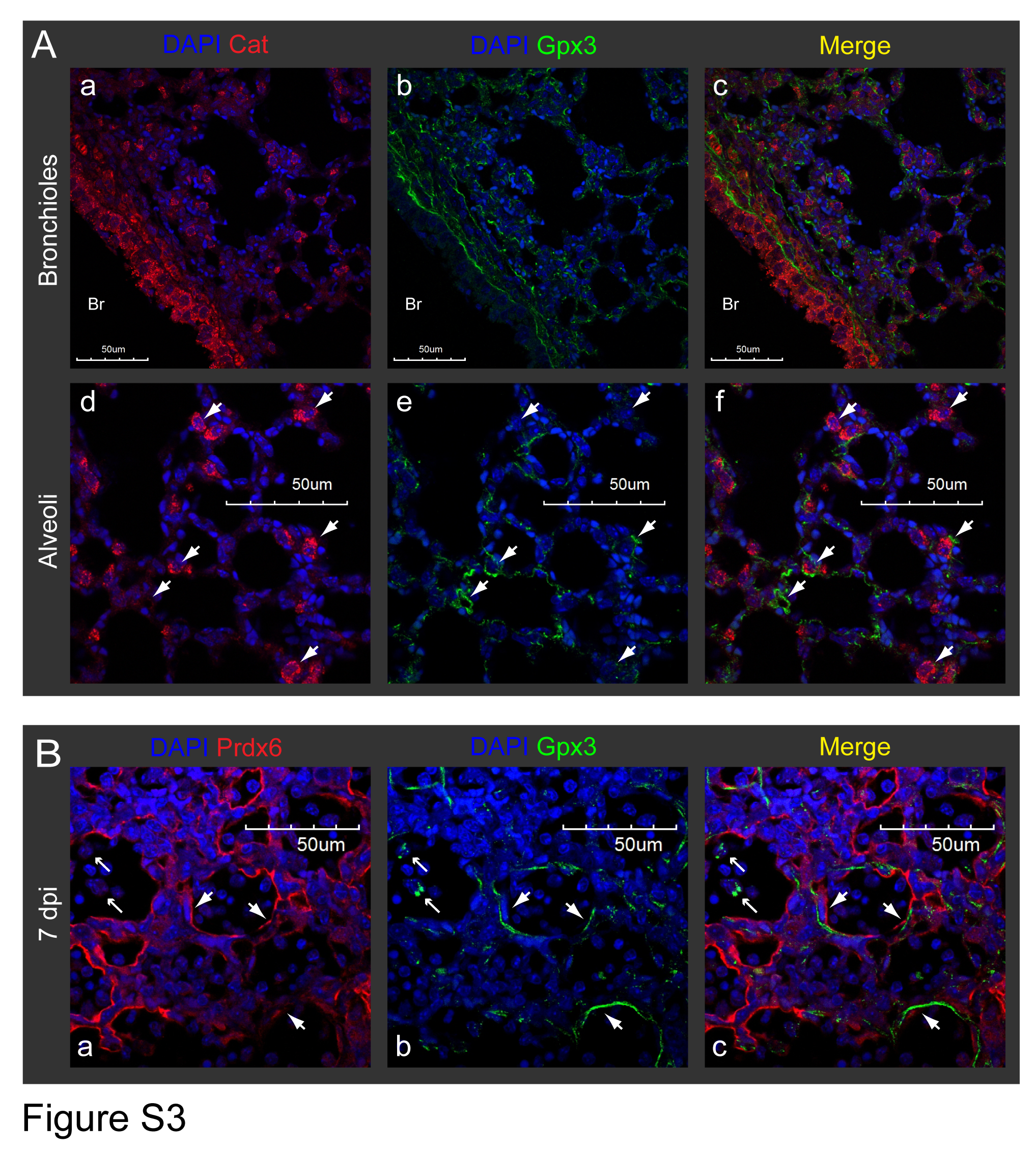

Supplement: Figure S3 — Localization of Gpx3 in both uninfected and infected mice lung. Mice were infected with sub-lethal dose of PR8 by intra-tracheal inhalation. Immunostained lung sections with the indicated antibodies and observed at ×60 magnification with confocal microscope. The scale bars represent 50 µm. A. Gpx3 was abundantly located at the basement membrane of blood vessels (Bv) and bronchioles (Br) in the normal lung. Gpx3 was also weakly detected at alveolus, but did not always appear at the surface of AT2 cells that express higher Cat (white arrows in d, e, f). B. Alveolar GPX3 at 7 dpi did not co-localized with AT1 cells that express higher level of Prdx6 (thick arrows in a, b, c). Gpx3 was also strongly detected within some infiltrated cells (thin arrows in a, b, c). (TIF) [file pone.0031494.s003.tif]
