# Supplementary material for: Major Shifts in the Spatio-Temporal Distribution of Lung Antioxidant Enzymes during Influenza Pneumonia
Source: PLoS One. 2012 Feb 15;7(2):e31494. doi: 10.1371/journal.pone.0031494 (PMC3280306; doi:10.1371/journal.pone.0031494)
Supplement: Supplementary file 2 [file pone.0031494.s004.doc]

Table S1. Primary antibodies used in this study.

Target	Company	Product No.	Host	Application in lung tissue		
				IHC-P (antigen retrieval)	IHC-Fr	WB
Actb	Sigma Aldrich	A5441	Mouse			+
Cat	Abcam	ab1877	Rabbit	+ (Proteinase K)		+
CCSP	Santa cruz	sc-9772	Goat	+ (Proteinase K)		+
CD68	Abcam	ab53444	Rat		+	
Gpx3	R&D systems	AF4199	Goat	+ (Proteinase K)		+
Hmox1	Enzo Life Sciences	ADI-SPA-896	Rabbit			+
Influenza M2	Santa cruz	sc-32238	Mouse			+
Influenza NS1	Santa cruz	sc-130568	Mouse	+ (Proteinase K)		
Pdpn	R&D systems	AF3244	Goat	+ (Proteinase K)		+
Prdx6	Abcam	ab59543	Rabbit	+ (Proteinase K)		+
Sod3	R&D systems	AF4817	Goat			+
SPC	Santa cruz	sc-7706	Goat	+ (Proteinase K)		
Tf	Santa cruz	sc-22599	Goat			+
TTF-1	Santa cruz	sc-13040	Rabbit			+
