# Supplementary material for: Major Shifts in the Spatio-Temporal Distribution of Lung Antioxidant Enzymes during Influenza Pneumonia
Source: PLoS One. 2012 Feb 15;7(2):e31494. doi: 10.1371/journal.pone.0031494 (PMC3280306; doi:10.1371/journal.pone.0031494)
Supplement: Supplementary file 3 [file pone.0031494.s005.doc]

Table S2. Primers used for qRT-PCR in this study.

Type	Gene ID	GeneBank accession	Primer	Primer sequences	Amplicon size (bp)	Source*
Beta actin	Actb	NM_007393	Forward	GGCTGTATTCCCCTCCATCG	154	ID 6671509a1
			Reverse	CCAGTTGGTAACAATGCCATGT		
Influenza	H1N1 NP	NC_002019	Forward	GGGTGAGAATGGACGAAAAA	97	[32]
			Reverse	TCCATCATTGCTTTTTGTGC		
Pro-inflammatory cytokine	Tnf	NM_013693	Forward	GGGACAGTGACCTGGACTGT	77	[32]
			Reverse	GCTCCAGTGAATTCGGAAAG		
Lung-abundant antioxidants	Cat	NM_009804	Forward	AGCGACCAGATGAAGCAGTG	181	ID 6753272a1
			Reverse	TCCGCTCTCTGTCAAAGTGTG		
	Gpx3	NM_008161	Forward	TGGCTTGGTCATTCTGGGC	103	ID 15011841a2
		NM_001083929	Reverse	CCCACCTGGTCGAACATACTT		
	Gpx8	NM_027127	Forward	CCTTTCGCTGCCTACCCATTA	140	ID 21312336a1
			Reverse	GAGTAGAAGCTGTTGGTTCTCG		
	Gstk1	NM_029555	Forward	GGTCCTATGCAGATACCAACAC	124	ID 21313138a1
			Reverse	GTACTGGCCTTTTCGGGGAA		
	Prdx6	NM_007453	Forward	CGCCAGAGTTTGCCAAGAG	116	ID 6671549a1
			Reverse	TCCGTGGGTGTTTCACCATTG		
	Sod3	NM_011435	Forward	CCTTCTTGTTCTACGGCTTGC	142	ID 6755600a1
			Reverse	TCGCCTATCTTCTCAACCAGG		
	Tf	NM_133977	Forward	GCTGTCCCTGACAAAACGGT	96	ID 20330802a1
			Reverse	CGGAAGGACGGTCTTCATGTG		
Macrophage-abundant antioxidants	Hmox1	NM_010442	Forward	AAGCCGAGAATGCTGAGTTCA	100	ID 6754212a1
			Reverse	GCCGTGTAGATATGGTACAAGGA		
	Ptgs2	NM_011198	Forward	TGAGCAACTATTCCAAACCAGC	74	ID 31981525a1
			Reverse	GCACGTAGTCTTCGATCACTATC		
CCSP	Scgb1a1	NM_011681	Forward	ATGAAGATCGCCATCACAATCAC	135	ID 6755947a1
			Reverse	GGATGCCACATAACCAGACTCT		
PDPN	Pdpn	NM_010329	Forward	ACCGTGCCAGTGTTGTTCTG	159	ID 6754042a1
			Reverse	AGCACCTGTGGTTGTTATTTTGT		
SPC	Sftpc	NM_011359	Forward	ACCATCGCTACCTTTTCCATCG	139	ID 6755480a3
			Reverse	CCTCAAGACTAGGGATGCTCTC		
TTF-1	Nkx2-1	NM_009385	Forward	GCTCGAGCGACGTTTCAAG	86	This study
		NM_001146198	Reverse	GCGTGGGTGTCAGGTGAATC		
F4/80	Emr1	NM_010130	Forward	CCCCAGTGTCCTTACAGAGTG	103	ID 33859546a1
			Reverse	GTGCCCAGAGTGGATGTCT		

*Primer sequences were obtained from PrimerBank (http://pga.mgh.harvard.edu/primerbank/index.html).
